# Impact of Preoperative Glucagon‐Like Peptide‐1 Receptor Agonist on Outcomes Following Major Surgery

**DOI:** 10.1002/wjs.12484

**Published:** 2025-01-09

**Authors:** Zayed Rashid, Selamawit Woldesenbet, Mujtaba Khalil, Abdullah Altaf, Jun Kawashima, Khalid Mumtaz, Timothy M. Pawlik

**Affiliations:** ^1^ Department of Surgery The Ohio State University Wexner Medical Center and James Comprehensive Cancer Center Columbus Ohio USA; ^2^ Department of Internal Medicine Division of Palliative Care The Ohio State University Wexner Medical Center and James Comprehensive Cancer Center Columbus Ohio USA

**Keywords:** GLP‐1RA, ileus, major surgical procedures, MarketScan, surgical complications

## Abstract

**Background:**

Glucagon‐like peptide‐1 receptor agonists (GLP‐1RA) are increasingly being used for the management of diabetes mellitus and obesity. We sought to define the impact of preoperative GLP‐1RA use on outcomes following major surgical procedures.

**Methods:**

Patients who underwent a major surgical procedure between 2013 and 2021 were identified using the IBM MarketScan database. Patients who took GLP‐1RA within a year before surgery were categorized as “exposed.” After propensity score matching (PSM), multivariable regression analysis was used to define the association of GLP‐1RA exposure with postoperative complications.

**Results:**

Among 138,980 patients (coronary artery bypass graft: *n* = 39,516, 28.4%; pneumonectomy: *n* = 4,881, 3.5%; abdominal aortic aneurysm repair: 4,459, 3.3%; pancreatectomy: *n* = 15,873, 11.4%; and colectomy: *n* = 74,251, 53.4%), most individuals were male (*n* = 80,871, 58.2%) with a median age of 58 (IQR 53–61) years. 2944 (2.2%) individuals had GLP‐1RA exposure before surgery. Overall incidence of complications was 36.5% (*n* = 50,724); complications included sepsis (*n* = 6,385, 4.6%), surgical site infections (*n* = 7,431, 5.3%), thromboembolism (*n* = 3,609, 2.6%), pneumonia (*n* = 4,783, 3.4%), renal (*n* = 9,017, 6.5%), or cardiopulmonary failure (*n* = 26,661, 19.2%). On unmatched analysis, patients on GLP‐1RA had a higher risk of complications (no GLP‐1RA: 36.3% vs. GLP‐1RA: 44.5% *p* < 0.001); however, after PSM to account for measured confounders, GLP‐1RA exposure was not associated with the odds of surgical complications (OR 0.99 95% CI 0.91–1.08; *p* > 0.05). Among patients using GLP‐1RA during the 2 weeks before surgery (*n* = 522, 17.7%), there was no association of GLP‐1RA with risk of complications (nonrecent GLP‐1RA: 44.7% vs. recent GLP‐1RA: 44.1%; *p* = 0.992).

**Conclusions:**

GLP‐1RA use was not associated with an increased risk of complications following major surgical procedures.

## Graphical abstract

GLP‐1RA use was not associated with an increased risk of complications following major surgical procedures (coronary artery bypass graft, pneumonectomy, abdominal aortic aneurysm repair, pancreatectomy, and colectomy) even among patients who continued the medication up until the time of surgery.

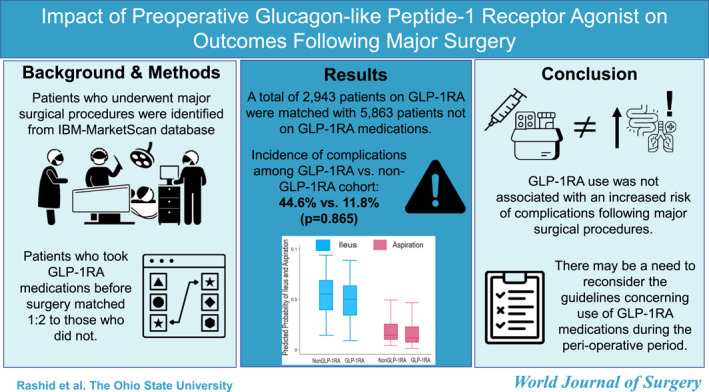

## 1 Introduction

Glucagon‐like peptide‐1 receptor agonists (GLP‐1RA) are increasingly being used for the management of diabetes mellitus (DM) and obesity [1, 2, 3]. The increased utilization can be attributed to the beneficial effects on weight loss as well as cardiovascular, renal, and liver function [1, 4]. However, these favorable outcomes can come at the expense of adverse effects, particularly gastrointestinal issues such as delayed gastric emptying [5]. In fact, up to 70% of individuals using GLP‐1RA may experience some type of adverse effect [5]. These undesirable effects can be particularly problematic among surgical patients, who are already susceptible to complications such as decreased gut motility, hypoglycemia, and aspiration [3]. Whether certain antidiabetic drugs, such as GLP‐1RA, contribute to the burden of surgical morbidity remains a topic of debate [3, 6]. In turn, there has been a growing interest in the evaluation of GLP‐1RA's impact on patients in the perioperative period [7].

Recent studies have noted that GLP‐1RA medications, traditionally associated with glucose regulation, can affect different physiological pathways [8, 9]. For example, the nonglycemic benefits of GLP‐1RA include weight loss, improved blood pressure regulation, enhanced cardiac function, and better management of dyslipidemia [1, 8, 9]. Therefore, the impact of GLP‐1RA drugs on medical comorbidities may indirectly benefit patients who are undergoing surgery [10, 11]. To this point, Buddhiraju et al. reported that GLP‐1RA use before elective total knee and hip replacement was associated with lower odds of postsurgical infection and readmission [6]. In a separate study, Dixit et al. reported no difference in respiratory complications relative to GLP‐1RA exposure among patients undergoing emergency gynecologic or orthopedic procedures [7]. In fact, some data have suggested that discontinuation of these medications during the perioperative period may worsen glycemic control and hinder recovery from surgery [12, 13].

Current guidelines from the American Society of Anesthesiologists (ASA) recommend stopping GLP‐1RA medications prior to surgery [14]. Withholding these newer agents of antiglycemic agents can lead to unnecessary delays in care and logistical challenges [7]. Given the limited data on the risks versus benefits of using GLP‐1RA in the perioperative period, we sought to define the impact of GLP‐1RA use on complications among patients undergoing a major surgical procedure.

## 2 Material and Methods

**FIGURE 1:**
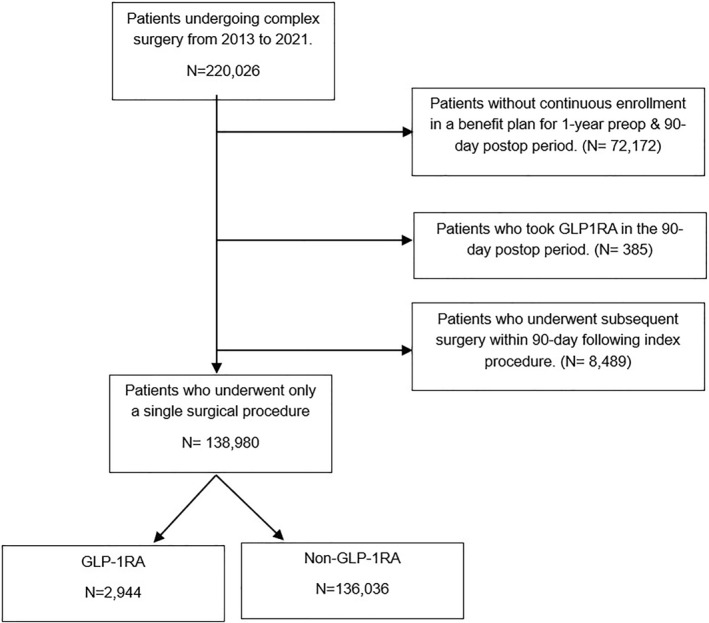
The flowchart depicting the number of patients included according to the study criteria.

Patients who underwent a major surgical procedure between 2013 and 2021 were identified from the commercial IBM MarketScan database (Supplementary eMethods in Supporting Information S1) [15]. The International Classification of Diseases, ninth and tenth editions (ICD‐9/10) codes were utilized to identify patients under 65 years of age who underwent coronary artery bypass graft (CABG), pneumonectomy, abdominal aortic aneurysm repair (AAA), pancreatectomy, and colectomy. National drug codes were used to identify patients who had at least one claim for GLP‐1RA therapy (Semaglutide, Albiglutide, Liraglutide, Dulaglutide, Lixisenatide, or Exenatide) from 1 year up to 15 days before the index surgery (i.e., “exposed”); the remaining cohort was designated as the non‐GLP‐1RA group (i.e., “non‐exposed”). Patients who had noncontinuous enrollment in the benefit plan for 1 year before and 90 days after surgery, had *de novo* GLP‐1RA initiation within 2 weeks before surgery or who took GLP‐1RA within 90 days after surgery were excluded (Figure 1). Similarly, patients who underwent an additional surgical procedure unrelated to the index procedure of interest were excluded from the study [16]. The need for informed consent for deidentified data was waived by the institutional review board (IRB) of the Ohio State University. This study followed the Strengthening the Reporting of Observational Studies in Epidemiology (STROBE) reporting guidelines.

### 2.1 Variables, Exposure, and Outcome

Variables of interest included patient age, sex, benefit plan type, employment status, Charlson Comorbidity Index (CCI), US census region (Northeast, Northcentral, South, and West), rurality (metropolitan vs. nonmetropolitan), claim‐based frailty index, and baseline concurrent medical conditions (Table 1). Patients were identified as residing in metropolitan or nonmetropolitan areas using the United States Department of Agriculture Rural‐Urban Continuum Codes [17, 18]. Patient sex was defined as male or female, based on sex assigned at birth [19]. Patient employment status was categorized as actively employed, retired, and “Other.” The “Other” category included individuals with long‐term disabilities, COBRA continuers, and dependents. Similarly, using the claim‐based frailty index, patients were categorized as nonfrail, prefrail, and frail [20]. Additionally, the CCI classified patients based on the severity of their preoperative comorbidities using a cutoff value of 2 as described in earlier studies [21, 22]. Furthermore, the ICD 9/10 codes identified any baseline comorbid conditions related to the cardiovascular, central nervous, and gastrointestinal systems (Table 2) [1].

**TABLE 1:** Baseline characteristics of patients who underwent complex surgical procedures relative to glucagon‐like peptide‐1 receptor agonists initiation.

Patient characteristics		Unmatched			Matched		
	Total *N* = 138,980	GLP‐1RA *N* = 2944	Non GLP‐1RA *N* = 136,036	*p*‐value	GLP‐1RA *N* = 2943	Non GLP‐1RA *N* = 5863	*p*‐value
Age, years							
Mean (SD)	54.9 ± 7.9	56.4 ± 6.2	53.4 ± 9.5	< 0.001	56.4 ± 6.2	56.4 ± 6.6	0.808
Median (IQR)	58 (53–61)	56 (49–60)	58 (53–61)	< 0.001	58 (53–61)	58 (53–61)	0.808
Sex							
Male	80,871 (58.2%)	1792 (60.9)	79,079 (58.1)	0.003	1151 (39.1)	2326 (39.7)	0.610
Female	58,109 (41.8%)	1152 (39.1)	56,957 (41.9)	0.003	1792 (60.9)	3537 (60.3)	0.610
Region							
Northeast	23,463 (16.9%)	373 (12.7)	23,090 (17.0)	< 0.001	373 (12.7)	727 (12.4)	0.780
North central	30,586 (22.0%)	574 (19.5)	30,012 (22.1)	< 0.001	574 (19.5)	1103 (18.8)	0.780
South	66,000 (47.5%)	1713 (58.2)	64,287 (47.3)	< 0.001	1712 (58.2)	3463 (59.1)	0.780
West	17,518 (12.6%)	271 (9.2)	17,247 (12.7)	< 0.001	271 (9.2)	535 (9.1)	0.780
Type of health insurance							
PPO	79,839 (57.4%)	1695 (57.6)	78,144 (57.4)	0.075	1694 (57.6)	3400 (58.0)	0.961
HMO	15,318 (11.0%)	280 (9.5)	15,038 (11.1)	0.075	280 (9.5)	535 (9.1)	0.961
Comprehensive	5694 (4.1%)	120 (4.1)	5574 (4.1)	0.075	120 (4.1)	228 (3.9)	0.961
POS	9788 (7.0%)	214 (7.3)	9574 (7.0)	0.075	214 (7.3)	435 (7.4)	0.961
Other^a^	28,341 (20.4%)	635 (21.6)	27,706 (20.4)	0.075	635 (21.6)	1265 (21.6)	0.961
Employment status							
Other	73,736 (53.1%)	1738 (59.0)	71,998 (52.9)	< 0.001	1735 (59.0)	3462 (59.0)	0.999
Full/part‐time	16,358 (11.8%)	389 (13.2)	15,969 (11.7)	< 0.001	389 (13.2)	775 (13.2)	0.999
Retired	48,886 (35.2%)	817 (27.8)	48,069 (35.3)	< 0.001	817 (27.8)	1626 (27.7)	0.999
CCI							
≤ 2	81,675 (58.8%)	1278 (43.4)	80,397 (59.1)	< 0.001	1278 (43.4)	2557 (43.6)	0.867
> 2	57,305 (41.2%)	1666 (56.6)	55,639 (40.9)	< 0.001	1665 (56.6)	3306 (56.4)	0.867
Claim‐based frailty index							
Nonfrail	90,213 (64.9%)	1044 (35.5)	89,169 (65.5)	< 0.001	1044 (35.5)	2093 (35.7)	0.925
Prefrail	47,284 (34.0%)	1840 (62.5)	45,444 (33.4)	< 0.001	1839 (62.5)	3657 (62.4)	0.925
Frail	1483 (1.1%)	60 (2.0)	1423 (1.0)	< 0.001	60 (2.0)	113 (1.9)	0.925
Rurality							
Metro	118,016 (84.9%)	2412 (81.9)	115,604 (85.0)	< 0.001	2411 (81.9)	4853 (82.8)	0.322
Nonmetro	20,964 (15.1%)	532 (18.1)	20,432 (15.0)	< 0.001	532 (18.1)	1010 (17.2)	0.322
Procedure							
AAA	4459 (3.3%)	77 (2.6)	4382 (3.2)	< 0.001	77 (2.6)	141 (2.4)	0.970
CABG	39,516 (28.4%)	1627 (55.3)	37,889 (27.9)	< 0.001	1627 (55.3)	3247 (55.4)	0.970
Colectomy	74,251 (53.4%)	935 (31.8)	73,316 (53.9)	< 0.001	935 (31.8)	1875 (32.0)	0.970
Pneumonectomy	4881 (3.5%)	101 (3.4)	4780 (3.5)	< 0.001	101 (3.4)	192 (3.3)	0.970
Pancreatectomy	15,873 (11.4%)	204 (6.9)	15,669 (11.5)	< 0.001	203 (6.9)	408 (7.0)	0.970

Abbreviations: AAA, abdominal aortic aneurysm; CABG, coronary artery bypass graft; CCI, Charlson Comorbidity Index; GLP‐1RA, glucagon‐like peptide‐1 receptor agonist; HMO, health maintenance organization; IQR, interquartile range; metro, metropolitan; POS, point of service plan; PPO, preferred provider organization; and SD, standard deviation.

^a^ Including, COBRA, long‐term disability, surviving spouse/depend, or other/unknown.

**TABLE 2:** Additional clinical characteristics of patients who underwent complex surgical procedures relative to glucagon‐like peptide‐1 receptor agonists initiation.

Patient characteristics		Unmatched			Matched		
	Total *N* = 138,980	GLP‐1RA *N* = 2944	Non GLP‐1RA *N* = 136,036	*p*‐value	GLP‐1RA *N* = 2943	Non GLP‐1RA *N* = 5863	*p*‐value
Myocardial infarction	17,223 (12.4%)	584 (19.8)	16,639 (12.2)	< 0.001	584 (19.8)	1121 (19.1)	0.418
Congestive heart failure	13,480 (9.7%)	619 (21.0)	12,861 (9.5)	< 0.001	619 (21.0)	1232 (21.0)	0.418
Peripheral vascular disease	4603 (3.3%)	103 (3.5)	4500 (3.3)	0.567	103 (3.5)	226 (3.9)	0.408
Cerebrovascular disease	8169 (5.9%)	319 (10.8)	7850 (5.8)	< 0.001	319 (10.8)	640 (10.9)	0.913
Dementia	90 (0.1%)	3 (0.1)	87 (0.1)	0.423	3 (0.1)	3 (0.1)	0.807
Hemiplegia or paraplegia	685 (0.5%)	20 (0.7)	665 (0.5)	0.144	20 (0.7)	44 (0.8)	0.712
Diabetes mellitus type 1	1087 (0.8%)	43 (1.5)	1044 (0.8)	< 0.001	43 (1.5)	78 (1.3)	0.619
Diabetes mellitus type 2	35,997 (25.9%)	2844 (96.6)	33,153 (24.4)	< 0.001	2843 (96.6)	5663 (96.6)	0.974
Hypertension	62,736 (45.1%)	1910 (64.9)	60,826 (44.7)	< 0.001	1909 (64.9)	3841 (65.5)	0.548
Obesity	8744 (6.3%)	497 (16.9)	8247 (6.1)	< 0.001	496 (16.9)	975 (16.6)	0.790
Dyslipidemia	45,278 (32.6%)	1576 (53.5)	43,702 (32.1)	< 0.001	1575 (53.5)	3082 (52.6)	0.400
Chronic renal disease	5394 (3.9%)	198 (6.7)	5196 (3.8)	0.005	198 (6.7)	417 (7.1)	0.504
Chronic pulmonary disease	19,955 (14.4%)	348 (11.8)	19,607 (14.4)	< 0.001	23 (0.8)	50 (0.9)	0.728
Peptic ulcer disease	927 (0.7%)	18 (0.6)	909 (0.7)	0.708	18 (0.6)	29 (0.5)	0.477
Rheumatic disease	1929 (1.4%)	23 (0.8)	1906 (1.4)	0.005	23 (0.8)	50 (0.9)	0.728
AIDS/HIV	285 (0.2%)	4 (0.1)	281 (0.2)	0.402	4 (0.1)	17 (0.3)	0.162
Any malignancy	37,712 (27.1%)	598 (20.3)	37,114 (27.3)	< 0.001	597 (20.3)	1199 (20.5)	0.856
Metastatic solid tumor	13,515 (9.7%)	193 (6.6)	13,322 (9.8)	< 0.001	428 (7.3)	193 (6.6)	0.200
Combination drugs	1353 (1.0%)	189 (6.4)	1164 (0.9)	< 0.001	189 (6.4)	364 (6.2)	0.697
Metformin	1196 (0.9%)	174 (5.9)	1022 (0.8)	< 0.001	174 (5.9)	353 (6.0)	0.840
SGLT‐2	904 (0.7%)	272 (9.2)	632 (0.5)	< 0.001	271 (9.2)	471 (8.0)	0.061
DPP‐4	1984 (1.4%)	200 (6.8)	1784 (1.3)	< 0.001	200 (6.8)	426 (7.3)	0.418
Sulphonylureas	1090 (0.8%)	115 (3.9)	975 (0.7)	< 0.001	115 (3.9)	212 (3.6)	0.495
Thiazolidinedione	318 (0.2%)	63 (2.1)	255 (0.2)	< 0.001	63 (2.1)	118 (2.0)	0.690
Statins	3231 (2.3%)	115 (3.9)	3116 (2.3)	< 0.001	115 (3.9)	228 (3.9)	0.966
Mild liver disease	5957 (4.3%)	151 (5.1)	5806 (4.3)	0.023	151 (5.1)	315 (5.4)	0.633
Severe liver disease	503 (0.4%)	12 (0.4)	491 (0.4)	0.677	12 (0.4)	21 (0.4)	0.720
NAFLD	3329 (2.4%)	113 (3.8)	3216 (2.4)	< 0.001	113 (3.8)	226 (3.9)	0.972
ALD	2582 (1.9%)	48 (1.6)	2534 (1.9)	0.356	48 (1.6)	103 (1.8)	0.668

Abbreviations: AIDS, acquired immunodeficiency syndrome; ALD, alcohol‐associated liver disease; DPP‐4, dipeptidyl peptidase‐4 inhibitors; GLP‐1RA, glucagon‐like peptide‐1 receptor agonist; HIV, human immunodeficiency virus; NAFLD, nonalcoholic fatty liver disease; and SGLT‐2, sodium‐glucose cotransporter‐2.

The primary outcome of interest was the development of complications within 30 days following surgery. The complications included sepsis, surgical site infections (SSIs), aspiration, hypoglycemia, pneumonia, ileus, venous thromboembolism (VTE), and acute respiratory, heart, or renal failure [7, 23, 24, 25, 26, 27]. Readmission occurring within the 30 days after the index surgery was a secondary outcome.

### 2.2 Statistical Analyses

Continuous variables were presented as median values with interquartile ranges (IQR) and were analyzed using either the Wilcoxon rank sum test or Student's t‐test as appropriate. Similarly, categorical variables were summarized as frequencies and percentages and were compared using either the chi‐squared test or Fisher's exact test [28]. Propensity score matching (PSM) was employed to account for measured confounders and reduce selection bias when comparing outcomes of patients who did and did not receive GLP‐1RA preceding surgery (Supplementary eMethods in Supporting Information S1). The patients were matched across baseline characteristics such as age, sex, region of residence, benefit plan type, employment status, CCI, claim‐based frailty index, rurality, type of surgical procedure, and other clinical characteristics outlined in Table 2. Following PSM, multivariable logistic regression models were employed to assess the association of GLP‐1RA exposure with outcomes; odds ratio (OR) with 95% confidence intervals (CIs) were reported. All statistical analyses were conducted using SAS 9.4 (SAS Institute) with statistical significance set at a *p*‐value of less than 0.05.

## 3 Results

### 3.1 Baseline Characteristics

A total of 138,980 patients underwent CABG (*n* = 39,516, 28.4%), pneumonectomy (*n* = 4,881, 3.5%), AAA repair (*n* = 4,459, 3.3%), pancreatectomy (*n* = 15,873, 11.4%), and colectomy (*n* = 74,251, 53.4%). Median age was 58 years (IQR: 53–61), most patients were male (*n* = 80,871, 58.2%), had a CCI score of ≤ 2 (*n* = 81,675, 58.8%), and were retirees (*n* = 48,886, 35.2%). Most patients were enrolled in a preferred provider organization plan (*n* = 79,839, 57.4%), followed by health maintenance organizations (*n* = 15,318, 11.0%), point of service plans (*n* = 9,788, 7.0%), and comprehensive plans (*n* = 5,694, 4.1%); the remainder were enrolled in other benefit plans (*n* = 28,341, 20.4%). Moreover, patients were primarily categorized as nonfrail (*n* = 90,213, 64.9%) and prefrail (*n* = 47,284, 34.0%), with only a few individuals categorized as frail (*n* = 1,483, 1.1%). Among other baseline characteristics, 25.9% (*n* = 35,997) of patients had a history of type 2 DM, 45.1% (*n* = 62,736) had hypertension (HTN), 32.6% (*n* = 45,278) had an abnormal lipid profile, 6.3% (*n* = 8744) were obese, and 27.1% (37,712) of individuals had a malignancy. Furthermore, 12.4% (*n* = 17,223) had a history of myocardial infarction, 9.7% (*n* = 13,480) had congestive heart failure, 5.9% (*n* = 8169) had cerebrovascular disease, and 2.4% (*n* = 3329) had nonalcoholic fatty liver disease (NAFLD).

Overall, 2.2% (*n* = 2944) of the patients were exposed to GLP‐1RA prior to surgery. Compared with patients who had no GLP‐1RA exposure, individuals exposed to GLP‐1RA were more likely to be female (41.9% vs. 39.1%), older (58 vs. 56 years), and retirees (35.3% vs. 27.8%) (all *p* < 0.05). Patients exposed to GLP‐1RA had a higher baseline comorbidity burden (CCI: 56.6% vs. 40.9%) and a higher claim‐based frailty (2.0 vs. 1.0) (both *p* < 0.001). The patterns of GLP‐1RA exposure also varied depending on the type of surgery (CABG: 55.3% vs. 27.9%; pneumonectomy: 3.4% vs. 3.5%; AAA repair: 2.6% vs. 3.2%; pancreatectomy: 6.9% vs. 11.5%; colectomy: 31.8% vs. 53.9%; *p* < 0.001). Perhaps not surprisingly, individuals with type 2 DM (96.6% vs. 24.4%), HTN (64.9% vs. 44.7%), obesity (16.9% vs. 6.1%), dyslipidemia (53.5% vs. 32.1%), and NAFLD (3.8% vs. 2.4%) were more likely to take GLP‐1RA (all *p* < 0.001). Similarly, patients with myocardial infarction (19.8% vs. 12.2%), congestive heart failure (21.0% vs. 9.5%), and cerebrovascular disease (10.8% vs. 5.8%) were also more likely to take GLP‐1RA (*p* < 0.001). In contrast, patients with a baseline malignancy (20.3% vs. 27.3%; *p* < 0.001) were less likely to be on GLP‐1RA before surgery. Additionally, patients who took GLP‐1RA had higher concurrent use of other antiglycemic medications such as sodium‐glucose cotransporter‐2 inhibitors (9.2% vs. 0.5%), dipeptidyl peptidase‐4 inhibitors (6.8% vs. 1.3%), sulphonylureas drugs (3.9% vs. 0.7%), and metformin (5.9% vs. 0.8%) (all *p* < 0.001).

Overall, 36.5% (*n* = 50,724) of patients experienced some type of complication in the postoperative period. The most common complications included acute heart failure (*n* = 14,494, 10.4%), ileus (*n* = 13,478, 9.7%), respiratory failure (*n* = 12,167, 8.8%), renal failure (*n* = 9,017, 6.5%), SSI (*n* = 7,431, 5.3%), sepsis (*n* = 6,385, 4.6%), pneumonia (*n* = 4,783, 3.4%), and VTE (*n* = 3,609, 2.6%). Other less common complications were aspiration (*n* = 625, 0.4%) and hypoglycemia (*n* = 283, 0.2%). In addition, 9.8% (*n* = 13,583) of patients were readmitted within 30 days following surgery.

**FIGURE 2:**
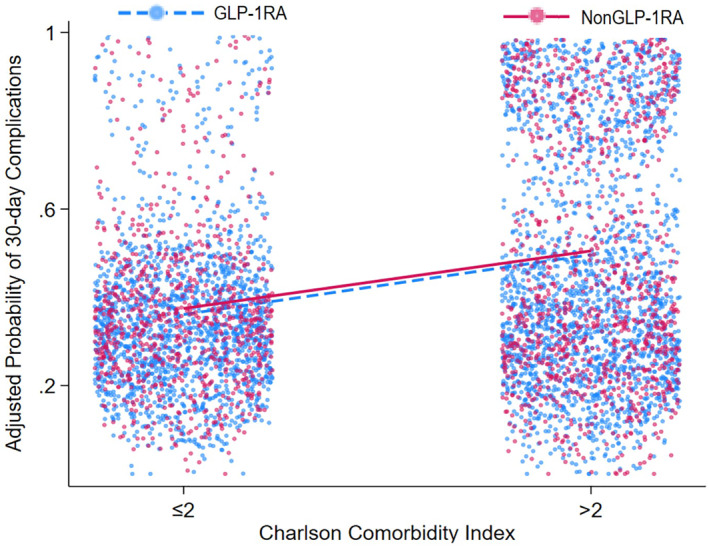
Adjusted risk of 30‐day complications following surgery relative to glucagon‐like peptide‐1 receptor agonist (GLP‐1RA) use.

**FIGURE 3:**
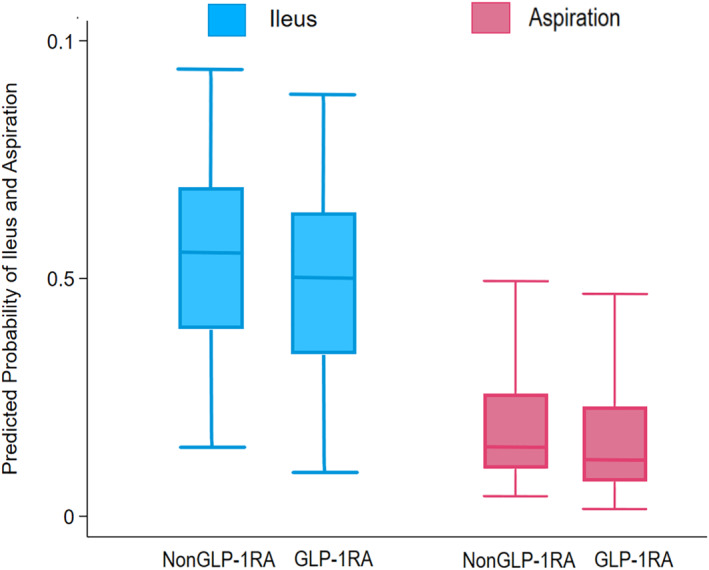
Boxplot depicting the adjusted probability of ileus and aspiration following complex surgical procedure relative to glucagon‐like peptide‐1 receptor agonist (GLP‐1RA) use.

After adjusting for measured confounders using PSM, cases were effectively matched with controls, which mitigated variations in baseline clinicodemographic characteristics (all *p* > 0.05) (Table 1). Subsequently, no differences were noted in the incidence of overall complications among GLP‐1RA users and nonusers (GLP‐1RA: 44.5% vs. no GLP‐1RA: 44.8%; *p* = 0.841) (Figure 2). Specifically, the incidence of sepsis (3.9% vs. 4.6%), SSI (4.2% vs. 5.4%), and hypoglycemia (0.2% vs. 0.1%) as well as most other complications was similar among patients who did and did not receive GLP‐1RA before surgery (all *p* > 0.05) (Table 3). Although there was a slight difference in the incidence of ileus (GLP‐1RA: 5.3% vs. no GLP‐1RA: 6.7%; *p* = 0.012) and aspiration (GLP‐1RA: 0.3% vs. no GLP‐1RA: 0.6%; *p* = 0.039), the clinical significance of these small differences was likely minimal (Figure 3). On multivariable analysis, GLP‐1RA exposure was not associated with higher odds of complications (OR 0.99 95% CI 0.91–1.08; *p* = 0.841) (Supplementary Table S1). There was also no difference in the likelihood of a 30‐day readmission (GLP‐1RA: 11.9% vs. no GLP‐1RA: 11.8%; *p* = 0.865). Additionally, stratified analyses were performed outlined in Supplementary Table S2.

**TABLE 3:** Outcomes of patients who underwent complex surgical procedures relative to glucagon‐like peptide‐1 receptor agonists initiation.

Outcomes		Unmatched			Matched		
	Total *N* = 138,980	GLP‐1RA *N* = 2944	Non GLP‐1RA *N* = 136,036	*p*‐value	GLP‐1RA	Non GLP‐1RA	*p*‐value
Complications	50,724 (36.5)	1311 (44.5)	49,413 (36.3)	< 0.001	1311 (44.6)	2625 (11.8)	0.865
Sepsis	6385 (4.6)	114 (3.9)	6281 (4.6)	0.056	114 (3.9)	268 (4.6)	0.130
SSI	7431 (5.3)	124 (4.2)	7307 (5.4)	0.006	124 (4.2)	259 (4.4)	0.658
Aspiration	625 (0.4)	8 (0.3)	617 (0.5)	0.145	8 (0.3)	35 (0.6)	0.039
Hypoglycemia	283 (0.2)	5 (0.2)	278 (0.2)	0.681	5 (0.2)	8 (0.1)	0.700
Ileus	13,478 (9.7)	157 (5.3)	13,321 (9.8)	< 0.001	157 (5.3)	393 (6.7)	0.012
Respiratory failure	12,167 (8.8)	410 (13.9)	11,757 (8.6)	< 0.001	410 (13.9)	795 (13.6)	0.632
Pneumonia	4783 (3.4)	138 (4.7)	4645 (3.4)	< 0.001	138 (4.7)	301 (5.1)	0.366
Acute heart failure	14,494 (10.4)	506 (17.2)	13,988 (10.3)	< 0.001	506 (17.2)	998 (17.0)	0.840
Acute renal failure	9017 (6.5)	354 (12.0)	8663 (6.4)	< 0.001	354 (12.0)	637 (10.9)	0.103
Venous thromboembolism	3609 (2.6)	91 (3.1)	3518 (2.6)	0.088	91 (3.1)	160 (2.7)	0.933
Readmission	13,583 (9.8)	350 (11.9)	13,233 (9.7)	< 0.001	350 (11.9)	690 (11.8)	0.865

Abbreviations: GLP‐1RA, glucagon‐like peptide‐1 receptor agonist; Postop, postoperative; and SSI, surgical site infection.

## 4 Discussion

Over the past decade, there has been an increased focus on improving the quality of surgical care and reducing postoperative complications [29, 30]. Consequently, there is a growing emphasis on understanding the preoperative and perioperative risk factors that may contribute to surgical morbidity [31]. Despite these efforts, complications particularly after major surgery continue to be a concern for both patients and clinicians [15, 31, 32]. In fact, roughly one in every four patient undergoing major surgery experiences a surgical complication [33]. Notably, medications used for comorbid conditions, such as DM, can differentially impact surgical outcomes [7, 34]. This is particularly true for newer drug classes that may be effective for one disorder but have complex interactions during transient nonhomeostatic states such as surgical episodes [35]. One such example is GLP‐1RA medications, which have recently gained popularity as a treatment of choice for multiple metabolic disorders [1, 2, 9]. Nonetheless, the association of GLP‐1RA with the incidence of postoperative complications remains a topic of debate [3, 6]. As such, the current study was important as it characterized the association of GLP‐1RA initiation status with outcomes following major surgical procedures. Notably, outcomes were comparable among the patients who did and did not receive GLP‐1RA before the surgical procedure. In fact, on stratified analysis, patients who received care compliant with American Society of Anesthesiologists guidelines and stopped GLP‐1RA before surgery had outcomes similar to individuals who continued using GLP‐1RA up until the time of surgery.

Previous studies have reported that GLP‐1RA drugs can slow down the gastrointestinal system either by directly binding to receptors or indirectly through modulation of insulin‐glucagon pathways [36, 37]. In fact, this is the primary mechanism behind increased satiety, which can decrease food consumption and consequently assist in both glycemic control improvement as well as weight reduction [37]. Nonetheless, there are concerns that this same mechanism of action can contribute to gastric content retention and increase complications related to anesthesia such as aspirations [38]. To this end, Yeo et al. reported that patients with GLP‐1RA usage had a 20% higher incidence of aspirations after endoscopic procedures [39]. In contrast, Dixit et al. demonstrated that among patients undergoing common emergency surgical procedures, 7.5% experienced short‐term respiratory complications, with no difference relative to GLP‐1RA utilization [39]. The current study noted that 10.9% of the patients experienced respiratory complications such as aspiration, pneumonia, or respiratory failure. Of note, there was no difference in the incidence of aspiration and pneumonia between the two groups, yet respiratory failure was slightly more common among patients taking GLP‐1RA. Nonetheless, after adjusting for measured confounders, this difference was almost completely mitigated. Similarly, the difference in the incidence of ileus following surgery relative to GLP‐1RA use was also clinically unremarkable. Odds of thromboembolism among patients with GLP‐1RA versus without GLP‐1RA exposure was also comparable (OR: 1.25 and 95% CI 0.87–1.79). In previous studies that used mice models, glucagon‐like peptide‐1 receptors had been demonstrated to cause inhibition of platelet aggregation in a nitric oxide‐dependent manner [40, 41]. Hence, some investigators had hypothesized that GLP‐1RA drugs may reduce thromboembolic events [40, 41].

Management of surgical patients can often be challenging, requiring optimization of metabolic profiles, particularly serum glucose levels [42, 43]. Blood sugar management is important among patients with or without DM because high glucose levels have been independently associated with higher mortality and morbidity [42, 43]. Among the morbidities attributed to poor glycemic control, sepsis and SSI are the most prominent [42, 44, 45]. To this point, one preclinical trial reported Semaglutide to be more effective in reducing bone or joint infections versus other therapies [46]. In another observational study, this association of GLP‐1RA use with infection risk was only noted among patients undergoing total hip replacement, while there was no benefit of GLP‐1RA in reducing SSI for total knee replacement [6]. In the current study, 30‐day risk of sepsis and SSI following a major surgical procedure was not associated with GLP‐1RA exposure. The lack of difference may be partly explained by the focus on major procedures in this study, for which more aggressive measures, such as close supervision and higher use of prophylactic antibiotics, are employed to prevent potentially severe infections [47].

According to ASA guidelines, it is recommended to discontinue GLP‐1RA before elective surgery [14]. For emergency procedures, in which timely discontinuation may not be possible, follow‐up with an abdominal ultrasound to check for gastric residual contents is advised [14]. In the current study, despite the vast majority of procedures being elective, one in every five patients continued taking GLP‐1RA until surgery. In addition, use of perioperative abdominal ultrasound was similar among both groups (Supplementary Table S2). These results highlight the variability in practices across different hospitals [48]. When weighing the achievement of optimal glycemic control along with potential multisystem benefits versus the risks of surgical complications that can or cannot occur, discontinuing the medication may not always be the preferred choice [12, 49]. To this end, some policymakers and healthcare associations have advocated liberalizing current guidelines to withhold GLP‐1RA prior to surgery [7].

The findings of the current studies should be interpreted in light of several limitations. Retrospective administrative datasets rely on ICD and national drug codes, which can be prone to inaccurate data input and missing values [50]. Therefore, there was a potential risk for misclassification bias and residual confounding. Additionally, the IBM MarketScan database is comprised of information on individuals and their dependents with employer‐sponsored benefit plans. In turn, the data may have limited generalizability to patients who are either uninsured or are enrolled in government‐sponsored health plans such as Medicare or Medicaid [19]. Additionally, as an observational study, the findings should be classified as associations rather than causations. However, the use of a robust statistical method of PSM did mitigate confounding by indication [51]. Data on granular patient‐level factors (e.g., race/ethnicity) or clinical characteristics (e.g., severity of comorbidities) that may influence the effects of GLP‐1RA could also not be assessed [52]. Despite these limitations, the study offers valuable insights into the association between GLP‐1RA and surgical outcomes, which would otherwise be difficult to investigate in clinical trials.

In conclusion, GLP‐1RA use was not associated with an increased risk of complications following major surgical procedures even among patients who continued the medication up until the time of surgery. There may be a need to reconsider the guidelines and liberalize the use of GLP‐1RA medications during the perioperative period.

## Author Contributions

**Zayed Rashid:** conceptualization, formal analysis, investigation, methodology, validation, visualization, writing–original draft, writing–review and editing. **Selamawit Woldesenbet:** conceptualization, data curation, formal analysis, investigation, methodology, software, validation, visualization, writing–original draft, writing–review and editing. **Mujtaba Khalil:** conceptualization, data curation, formal analysis, investigation, methodology, validation, writing–original draft, writing–review and editing. **Abdullah Altaf:** conceptualization, data curation, formal analysis, methodology, visualization, writing–original draft, writing–review and editing. **Jun Kawashima:** conceptualization, investigation, methodology, writing–original draft, writing–review and editing. **Khalid Mumtaz:** conceptualization, project administration, supervision, writing–original draft, writing–review & editing. **Timothy M. Pawlik:** conceptualization, investigation, methodology, project administration, resources, supervision, validation, visualization, writing–original draft, writing–review and editing.

## Ethics Statement

The need for informed consent for deidentified data was waived by the institutional review board of the Ohio State University.

## Conflicts of Interest

The authors declare no conflicts of interest.

## Supporting information

Supporting Information S1

## Data Availability

The data for this study were obtained from the IBM MarketScan database. There are restrictions to the availability of this data, which are used under license for this study. Data can be accessed with the permission from the IBM MarketScan Commercial Database.
