# Supplementary material for: Impact of Preoperative Glucagon‐Like Peptide‐1 Receptor Agonist on Outcomes Following Major Surgery
Source: World J Surg. 2025 Jan 9;49(3):698–707. doi: 10.1002/wjs.12484 (PMC11903251; doi:10.1002/wjs.12484)
Supplement: Supplementary file 1 — Supporting Information S1 [file WJS-49-698-s001.docx]

**Supplementary eMethods:**

*IBM-MarketScan database:*

This comprehensive national database is comprised of de-identified individual-level data from claim records across inpatient, outpatient, and prescription drug services for over 230 million privately insured enrollees and their dependents.^1^ The database also covers the Consolidated Omnibus Budget Reconciliation Act (COBRA) continuers, early retirees, and Medicare-eligible retirees with employer-provided Medicare Supplemental plans.^1^ The database has been used in previous clinical outcomes and epidemiological research to study the impact of therapeutics.^2–4^

*Propensity Score Matching:*

Patients were matched by clinicodemographic (e.g., sex, age, geographic region, CCI, rurality, type of benefit plan, type and year of surgical procedure, and concurrent use of other anti-glycemic medications), as well as baseline comorbidity status (e.g., diseases related to cardiovascular, pulmonary, gastric, renal, or central nervous system). The nearest neighbor “greedy” matching technique with a caliper width of 0.05 paired cases with controls.

*Stratified analyses*

After stratifying the GLP-1RA user group into patients who discontinued the medication two weeks before surgery versus patients who continued it until the day of surgery, there were no differences in outcomes (**Supplementary Table 2**). Similarly, stratified analysis by surgical case status (i.e., elective vs. emergent) demonstrated no difference in risk of complications relative to GLP-1RA use among patients who underwent an elective procedure (GLP-1RA: 76.4% vs. no GLP-1RA: 79.5%; p=0.436) versus patients who had an emergent operation (GLP-1RA: 83.3% vs. no GLP-1RA: 60.7%; p=0.162).

| **Supplementary Table 1:** Postoperative outcomes relative to GLP-1RA versus non-GLP-1RA | | | |
| --- | --- | --- | --- |
|  | **OR** | **95% CI** | **p-value** |
| Complications | 0.99 | 0.91 – 1.08 | 0.841 |
| Aspiration | 0.45 | 0.21 – 0.98 | 0.044 |
| Hypoglycemia | 1.25 | 0.41 – 3.81 | 0.700 |
| Ileus | 0.78 | 0.65 – 0.95 | 0.013 |
| Sepsis | 0.84 | 0.67 – 1.05 | 0.130 |
| SSI | 0.95 | 0.77 – 1.19 | 0.658 |
| Respiratory failure | 1.03 | 0.91 – 1.17 | 0.632 |
| Pneumonia | 0.91 | 0.74 – 1.12 | 0.366 |
| Acute heart failure | 1.01 | 0.90 – 1.14 | 0.840 |
| Acute renal failure | 1.12 | 0.98 – 1.29 | 0.103 |
| Venous thromboembolism | 1.14 | 0.88 – 1.48 | 0.334 |
| Readmission | 1.01 | 0.88 – 1.16 | 0.865 |
| ^GLP1-RA, glucagon-like peptide-1 receptor agonist; SSI: surgical site infections; Postop: postoperative; CI: confidence interval.^ | | | |

| **Supplementary Table 2:** Patients stratified based on GLP-1RA exposure within 2 weeks prior to surgery. | | | | |
| --- | --- | --- | --- | --- |
|  | **Total**  **(N=2,943)** | **GLP-1RA ≤ 2 weeks before surgery (N=522)** | **GLP-1RA > 2 weeks before surgery (N=2,421)** | **p-value** |
| Age |  |  |  |  |
| Mean±SD | 56.6±6.1 | 56.8±5.8 | 56.3±6.3 | 0.044 |
| Median (IQR) | 58 (53 – 61) | 58 (53 – 61) | 58 (53 – 61) | 0.044 |
| Sex |  |  |  |  |
| Female | 1,151 (39.1) | 174 (33.3) | 977 (40.4) | 0.003 |
| Male | 1,792 (60.9) | 348 (66.7) | 1,444 (59.6) | 0.003 |
| Frailty score |  |  |  |  |
| Non-frail | 1,044 (35.5) | 170 (32.6) | 874 (36.1) | 0.252 |
| Prefrail | 1,839 (62.5) | 339 (64.9) | 1,500 (62.0) | 0.252 |
| Frail | 60 (2.0) | 13 (2.5) | 47 (1.9) | 0.252 |
| Charlson comorbidity index |  |  |  |  |
| ≤2 | 1,278 (43.4) | 237 (45.4) | 1,041 (43.0) | 0.315 |
| >2 | 1,665 (56.6) | 285 (54.6) | 1,380 (57.0) | 0.315 |
| Type of health insurance |  |  |  |  |
| PPO | 1,694 (57.6) | 291 (55.7) | 1,403 (58.0) | 0.231 |
| HMO | 280 (9.5) | 50 (9.6) | 230 (9.5) | 0.231 |
| Comprehensive | 120 (4.1) | 14 (2.7) | 106 (4.4) | 0.231 |
| POS | 214 (7.3) | 43 (8.2) | 171 (7.1) | 0.231 |
| Other^a^ | 635 (2.2) | 124 (23.8) | 511 (21.1) |  |
| Employment status |  |  |  |  |
| Other | 1,737 (59.0) | 312 (59.8) | 1,425 (58.9) | 0.271 |
| Full/part-time | 389 (13.2) | 58 (11.1) | 331 (13.7) | 0.271 |
| Retired | 817 (27.8) | 152 (29.1) | 665 (27.5) | 0.271 |
| Region |  |  |  |  |
| Northeast | 373 (12.7) | 79 (15.1) | 294 (12.1) | 0.325 |
| North Central | 574 (19.5) | 92 (17.6) | 482 (19.9) | 0.325 |
| South | 1,712 (58.2) | 305 (58.4) | 1,407 (58.1) | 0.325 |
| West | 271 (9.2) | 44 (8.4) | 227 (9.4) | 0.325 |
| Rurality |  |  |  |  |
| Metro | 2,411 (81.9) | 432 (82.8) | 1,979 (81.7) | 0.585 |
| Non-Metro | 532 (18.1) | 90 (17.2) | 442 (18.3) | 0.585 |
| Procedure |  |  |  |  |
| AAA | 77 (2.6) | 15 (2.9) | 62 (2.6) | 0.001 |
| CABG | 1,627 (55.3) | 323 (61.9) | 1,304 (53.9) | 0.001 |
| Colon | 935 (31.8) | 145 (27.8) | 790 (32.6) | 0.001 |
| Pancreatectomy | 101 (3.4) | 5 (1.0) | 96 (4.0) | 0.001 |
| Pneumonectomy | 203 (6.9) | 34 (6.5) | 169 (7.0) | 0.001 |
| Covariates |  |  |  |  |
| Dyslipidemia | 1,575 (53.5) | 283 (54.2) | 1,292 (53.4) | 0.725 |
| Obesity | 496 (16.9) | 88 (16.9) | 408 (16.9) | 0.998 |
| Hypertension | 1,909 (64.9) | 314 (60.2) | 1,595 (65.9) | 0.013 |
| AIDS | 4 (0.1) | 1 (0.2) | 3 (0.1) | 0.704 |
| Congestive heart failure | 619 (21.0) | 116 (22.2) | 503 (20.8) | 0.462 |
| Chronic pulmonary disease | 348 (11.8) | 60 (11.5) | 288 (11.9) | 0.797 |
| Any malignancy | 597 (20.3) | 86 (16.5) | 511 (21.1) | 0.017 |
| Hemiplegia or paraplegia | 20 (0.7) | 2 (0.4) | 18 (0.7) | 0.363 |
| Renal disease | 198 (6.7) | 44 (8.4) | 154 (6.4) | 0.087 |
| Rheumatic disease | 23 (0.8) | 2 (0.4) | 21 (0.9) | 0.254 |
| Metastatic solid tumor | 193 (6.6) | 30 (5.7) | 163 (6.7) | 0.409 |
| Peptic ulcer disease | 18 (0.6) | 2 (0.4) | 16 (0.7) | 0.460 |
| Cardiovascular disease | 319 (10.8) | 57 (10.9) | 262 (10.8) | 0.948 |
| Peripheral vascular disease | 103 (3.5) | 21 (4.0) | 82 (3.4) | 0.473 |
| Diabetes mellitus type 1 | 45 (1.5) | 7 (1.3) | 36 (1.5) | 0.801 |
| Diabetes mellitus type 2 | 2,843 (96.6) | 517 (99.0) | 2,326 (96.1) | 0.001 |
| Mild Liver Disease | 151 (5.1) | 17 (3.3) | 134 (5.5) | 0.032 |
| Severe Liver Disease | 12 (0.4) | 4 (0.8) | 8 (0.3) | 0.156 |
| Myocardial infarction | 584 (19.8) | 112 (21.5) | 472 (19.5) | 0.309 |
| NAFLD | 113 (3.8) | 14 (2.7) | 99 (4.1) | 0.129 |
| ALD | 48 (1.6) | 11 (2.1) | 37 (1.5) | 0.344 |
| Combination drugs | 189 (6.4) | 38 (7.3) | 151 (6.2) | 0.378 |
| Sulphonylureas | 115 (3.9) | 26 (5.0) | 89 (3.7) | 0.163 |
| Metformin | 174 (5.9) | 32 (6.1) | 142 (5.9) | 0.816 |
| Statin | 115 (3.9) | 25 (4.8) | 90 (3.7) | 0.252 |
| DPP4 | 200 (6.8) | 30 (5.7) | 170 (7.0) | 0.294 |
| SGLT2 inhibitors | 271 (9.2) | 44 (8.4) | 227 (9.4) | 0.497 |
| Thiazolidinediones | 63 (2.1) | 14 (2.7) | 49 (2.0) | 0.346 |
| Complications | 1,311 (44.6) | 230 (44.1) | 1,081 (44.7) | 0.806 |
| Ileus | 157 (5.3) | 27 (5.2) | 130 (5.4) | 0.856 |
| Hypoglycemia | 5 (0.2) | 0 (0.0) | 5 (0.2) | 0.299 |
| Aspiration | 8 (0.2) | 2 (0.4) | 6 (0.2) | 0.590 |
| Sepsis | 114 (3.9) | 16 (3.1) | 98 (4.0) | 0.291 |
| SSI | 124 (4.2) | 15 (2.9) | 109 (4.5) | 0.093 |
| Respiratory failure | 410 (13.9) | 77 (14.8) | 333 (13.8) | 0.551 |
| Pneumonia | 138 (4.7) | 19 (3.6) | 119 (4.9) | 0.211 |
| Acute heart failure | 506 (17.2) | 93 (17.8) | 413 (17.1) | 0.678 |
| Acute renal failure | 354 (12.0) | 56 (10.7) | 298 (12.3) | 0.314 |
| Venous thromboembolism | 91 (3.1) | 16 (3.1) | 75 (3.1) | 0.969 |
| Readmission | 350 (11.9) | 60 (11.5) | 290 (12.0) | 0.757 |
| Postop abdominal USG | 66 (2.2) | 13 (2.5) | 53 (2.2) | 0.695 |
| ^GLP1-RA, glucagon-like peptide-1 receptor agonist; IQR, interquartile range; SD, standard deviation; CCI, Charlson Comorbidity index; AAA: abdominal aortic aneurysm; CABG: coronary artery bypass graft; metro: metropolitan; USG: ultrasound; SSI: surgical site infections; SGLT-2, sodium-glucose cotransporter-2; ALD: alcohol-associated liver disease; NAFLD: non-alcoholic fatty liver disease; DPP-4, dipeptidyl peptidase-4 inhibitors; AIDS, acquired immunodeficiency syndrome; PPO: preferred provider organization; HMO: health maintenance organization; POS: point of service plan; postop: postoperative.^  ^a Including, COBRA, Long-Term Disability, Surviving Spouse/Depend or Other/Unknown^ | | | | |

**References:**

1. Butler AM, Nickel KB, Overman RA, Brookhart MA. IBM MarketScan Research Databases. In: Sturkenboom M, Schink T, eds. *Databases for Pharmacoepidemiological Research*. Springer International Publishing; 2021:243-251. doi:10.1007/978-3-030-51455-6_20

2. Elsaid MI, Li N, Firkins SA, et al. Impacts of glucagon‐like peptide‐1 receptor agonists on the risk of adverse liver outcomes in patients with metabolic dysfunction‐associated steatotic liver disease cirrhosis and type 2 diabetes. *Aliment Pharmacol Ther*. 2024;59(9):1096-1110. doi:10.1111/apt.17925

3. Khalil M, Woldesenbet S, Munir MM, et al. Healthcare utilization and expenditures among patients with venous thromboembolism following gastrointestinal cancer surgery. *Journal of Gastrointestinal Surgery*. Published online May 2024:S1091255X24004517. doi:10.1016/j.gassur.2024.05.012

4. Endo Y, Woldesenbet S, Tsilimigras DI, et al. Effect of telemedicine use on medical spending and health care utilization among patients with gastrointestinal cancer. *Journal of Gastrointestinal Surgery*. Published online June 18, 2024. doi:10.1016/j.gassur.2024.06.009
